# Asthma and Sinonasal Diseases: Shared Clinical Comorbidities, Inflammatory Pathways and Management Strategies

**DOI:** 10.3390/medsci14050568

**Published:** 2026-09-14

**Authors:** Francesca Cefaloni, Enrico Schiavi, Matteo Morviducci, Eugenio De Corso, Marco Brunori, Simonetta Masieri, Matteo Bonini

**Affiliations:** 1 Department of Cardiovascular and Pulmonary Sciences, Università Cattolica del Sacro Cuore, 00168 Rome, Italy; 2 Department of Public Health and Infectious Diseases, Sapienza University of Rome, AOU Policlinico Umberto I, 00185 Rome, Italy; enricoschiavi@gmail.com (E.S.);; 3 Department of Public Health and Infectious Diseases, Sapienza University of Rome, 00185 Rome, Italy; 4 Department of Medicine & Surgery, Section of Otorhinolaryngology, Santa Maria Della Misericordia’s Hospital, University of Perugia, 06129 Perugia, Italy; 5 Department of Oral and Maxillo Facial Sciences, Sapienza University of Rome, 00185 Rome, Italy; 6 National Heart and Lung Institute (NHLI), Imperial College London, London SW7 2AZ, UK

**Keywords:** asthma, CRSwNP, biologics, precision medicine, comorbidities, inflammation

## Abstract

Asthma and sinonasal diseases, particularly chronic rhinosinusitis with nasal polyps (CRSwNP), frequently coexist as part of the United Airway Disease (UAD), reflecting shared inflammatory pathways and physiopathologic mechanisms. They both significantly contribute as additional comorbidities to patient burden of symptoms and disease severity, and may guide the treatment approach, particularly when presenting together. The aim of this review was to explore the clinical interplay between asthma and sinonasal diseases, emphasizing epidemiologic links, shared immunologic pathways, and emerging management strategies, namely biologic therapies targeting type 2 (T2) inflammation. This approach enables integrated diagnostic and therapeutic approaches by targeting specific inflammatory mediators in a precision-medicine perspective. This may lead to a higher chance of achieving better outcomes in both upper and lower airways. Future studies should refine the treatment selection and evaluate long-term effects of biologic therapy across the upper and lower airways.

## 1. Introduction

Asthma and sinonasal diseases, with particular regard to chronic rhinosinusitis (CRS), are frequently associated as part of the United Airway Disease (UAD) concept, which encompasses common inflammatory pathways across the upper and lower airways.

This review aims to highlight the epidemiologic, pathophysiological and clinical relationship between these two conditions, in particular the deep interaction between asthma and CRS with nasal polyposis (CRSwNP) characterised by T2 inflammation, as well as the contribution of additional comorbidities to patient burden of symptoms and concomitant treatment.

Ultimately, over the last few years, evidence from the literature has supported the role of monoclonal antibodies in both severe asthma and CRSwNP, with particular benefit in the case of overlapping diseases. Biologic therapies offer a precision-medicine approach, underscoring the need for a multidisciplinary diagnostic and therapeutic approach in this field.

We performed a PubMed search of free full text articles including metanalyses, randomised controlled trials (RCTs), and observational studies, from 2000 to 2026. We also included older articles outside this window due to their relevance on the topic. For Section 2 and Section 3 the research terms were “ASTHMA” or “BRONCHIAL ASTHMA” and “CRONIC RHINOSINUSITIS” or “COMORBIDITIES”.

## 2. Epidemiology of Comorbid Asthma and Sinonasal Diseases

Epidemiological studies consistently support the concept of United Airway Disease (UAD), confirming the strong and bidirectional association between asthma and sinonasal diseases, particularly allergic rhinitis (AR), CRS and CRSwNP [1,2].

The worldwide median prevalence of AR in adults is estimated at 18.1% [2,3]. Among asthmatic patients, the prevalence of AR is markedly higher, ranging between 30 and 80% [4]. Similarly, the prevalence of asthma is significantly higher in patients with AR than in the general population [4]. AR frequently anticipates the development of asthma, with an average interval of two years [2,5]. Moreover, the coexistence of AR and asthma is associated with more severe symptoms and a greater need for pharmacological treatment [6]. Interestingly, non-allergic rhinitis, despite the lack of an allergic phenotype, is also associated with an increased risk of developing asthma [7,8].

The prevalence of CRS across Europe ranges from 7% to 28%, with an estimated average of 11.9% [9], while the prevalence of CRSwNP in the general population is estimated to be between 1% and 4% [9,10]. The Global Allergy and Asthma European Network and other epidemiological studies with large cohorts have described asthma as the most frequent comorbidity in CRS, particularly in CRSwNP, in which asthma affects up to two-thirds of patients (range 27.0–66.7%) [9,10,11,12,13]. The UK National Chronic Rhinosinusitis Epidemiology Study described the prevalence of asthma among patients with different CRS phenotypes compared with controls [11]. The prevalence was 21.2% in CRS without nasal polyposis (CRSsNP), 46.9% in CRSwNP, and 73.3% in allergic fungal rhinosinusitis, compared with 10.0% in controls [11].

A large Korean epidemiological study including more than 200,000 patients with asthma and 30,000 patients with CRS, compared with matched controls, described a clear bidirectional relationship between the two diseases [14]. Specifically, patients with asthma had an increased risk of developing CRS (adjusted hazard ratio—aHR 1.74; 95% confidence interval—CI 1.67–1.80), CRSwNP (aHR 1.55, 95%CI 1.36–1.78) and CRSsNP (aHR 1.74, 95%CI 1.67–1.81). Similarly, patients with CRS had an increased risk of developing asthma (aHR 1.85, 95%CI 1.80–1.91), with similar results observed for both CRSwNP and CRSsNP phenotypes [14].

Another Korean epidemiological study involving nearly 18,000 patients described the relationship between asthma onset and CRS phenotypes [15]. In particular, CRSsNP was more frequently associated with childhood-onset (<18 years) and late-onset (>40 years) asthma, whereas CRSwNP was more frequently associated with early-onset (<40 years) asthma [15]. The relationship between CRS and asthma is clinically relevant, as their coexistence results in worse disease courses, characterised by poorer quality of life, more severe symptoms, higher exacerbation rates, and increased corticosteroid use [10,13,16]. Aspirin-exacerbated respiratory disease, present in approximately 7% of adults with asthma and up to 15% of those with severe asthma, forms a classic triad with asthma and CRS and further increases the severity of both conditions [17].

## 3. The Clinical Picture: In Common Comorbidities of Asthma and Sinonasal Diseases

The complex interplay between asthma and sinonasal diseases extends beyond the UAD concept [1,2]. Both conditions frequently coexist with respiratory and extra-pulmonary diseases, some of which may share inflammatory and/or mechanical pathways, contributing to disease severity, amplifying symptoms, and impairing disease control [18]. Therefore, these conditions should not be regarded merely as coincidental findings, but rather as potentially relevant treatable traits whose identification and appropriate management may improve overall disease control [1,2,18].

Among respiratory comorbidities, obstructive sleep apnoea (OSA) is one of the most prevalent and consistently associated with both asthma and CRS [19,20,21,22,23]. Patients with CRS have 1.76-fold higher odds of developing OSA, whereas asthmatic patients show a 2.6-fold increased risk [24]. Repetitive episodes of nocturnal hypoxia–reoxygenation and mechanical vibration of the upper airway during snoring promote systemic and local inflammation, ultimately leading to airway remodelling, bronchial hyperreactivity, and pharyngeal collapsibility [22]. OSA is associated with poorer asthma control, higher exacerbation rates, and reduced quality of life [22,23]. Conversely, asthma and CRS through their anatomical and inflammatory alterations of the upper airways may predispose OSA, establishing a bidirectional link between these disorders [22,23]. Notably, treatment with continuous positive airway pressure (CPAP) in patients with OSA has been shown to improve asthma control and reduce systemic inflammation [25].

The prevalence of vocal cord dysfunction (VCD) in asthma is estimated at 18%, and the condition is also frequently reported in patients with CRS [26,27]. It is characterized by inappropriate vocal cord adduction during inspiration [24,26,27]. Chronic airway inflammation and direct stimulus of post-nasal drip have been proposed as key mechanisms linking VCD with asthma and CRS [24,26]. Similarly, dysfunctional breathing is another common but frequently underdiagnosed comorbidity in asthma [26,27], whereas its association with sinonasal diseases remains unclear.

Chronic obstructive pulmonary disease (COPD) is strongly associated with asthma (odds ratio 6.23), while its association with CRS is weaker (odds ratio 1.73) [13,27]. The coexistence of asthma and COPD markedly worsens the disease course and increases mortality [20]. Current evidence supports a model in which T2-low inflammation—driven by smoking, air pollution or other environmental determinants in a genetically predisposed subject—may lead to fixed airway obstruction and COPD in a patient with asthma [28,29]. Conversely, in atopic individuals, COPD-driven inflammation may trigger T2 inflammation and asthma [28,30]. The pathophysiological link between CRS and COPD is currently unknown, but data suggest common etiological mechanisms such as oxidative stress and systemic inflammation [21].

Bronchiectasis represents a chronic respiratory disease increasingly associated with asthma and CRS [31,32]. In a recent meta-analysis, bronchiectasis was strongly associated with asthma, with an odds ratio of 4.89, whereas its prevalence in CRS remains unknown [21,27,33]. The pathophysiological link between asthma, CRS, and bronchiectasis lies in the persistent eosinophilic inflammation, which promotes epithelial damage and impaired mucociliary clearance [2,31], facilitating repeated infections, subsequent neutrophilic inflammation, and progressive airway remodelling [31]. In addition, the inflamed paranasal sinuses can act as reservoirs of pathogenic bacteria [2,32]. These mechanisms enable a vicious circle that worsens asthma and CRS control [2,31,32].

Among extra-pulmonary comorbidities, 40–80% of asthmatic patients present symptoms related to gastroesophageal reflux (GERD) [21,24,27,34]. Patients with CRS also show a higher prevalence of GERD compared to healthy individuals [35]. Acid-related irritation and microaspiration in GERD can induce bronchoconstriction, injury to the sinonasal mucosa and CRS, potentially contributing to both asthma and CRS symptoms, and may promote or worsen GERD [21,24,27]. GERD represents an independent risk factor for exacerbations and is associated with worse asthma symptoms [24]. Comprehensive GERD management should always be considered in patients with asthma or CRS [21,24]. Proton pump inhibitors have shown variable efficacy in improving asthma control, whereas surgical interventions have yielded more promising results [24,36,37,38].

Metabolic syndrome is frequent in both asthma and CRS. The prevalence of asthma in obese patients is estimated to exceed 50%, while patients with type II diabetes mellitus (DMII) have a 43% increased risk of developing asthma [27]. Obesity shows a moderate association with both asthma and CRS, and a strong association with severe asthma (odds ratio 4.06) [21,27,33,39]. Adipocytes secrete proinflammatory adipokines which increase systemic inflammation by generating reactive oxygen species, activating natural killer cells, enhancing macrophage activity—especially M1 macrophages—and promoting cytokine release [21,27]. Moreover, obesity may alter the expression of genes that regulate glucocorticoid receptors, leading to a reduced responsiveness to glucocorticoid therapy [21,27]. The pathophysiological link between DMII and asthma remains unclear, whereas its association with CRS may be explained by hyperglycaemia-induced impairment of innate immune responses, leading to higher fungal and bacterial colonization rates [21,27].

Patients with asthma and CRS have higher odds of having both anxiety and depression [21,27,40]. The pathophysiological link underlying this association involves serotonin dysregulation, systemic inflammation, and genetic or environmental susceptibility, while corticosteroid neurotoxicity may further contribute [21,27].

Lastly and particularly relevant, T2 non-respiratory diseases, such as atopic dermatitis and eosinophilic esophagitis, are frequently associated both with CRS and asthma [41]. These diseases share a common T2-driven pathophysiology and are targeted by the same group of biologic treatments [41], as detailed below.

Although the mechanisms underlying these associations are heterogeneous and not fully elucidated, comorbidities may influence asthma and CRS through multiple and often overlapping pathways, including local or systemic inflammation, altered airway mechanics, symptom amplification, and impaired treatment response. Their coexistence may therefore contribute to the overall disease burden beyond the effect of each individual condition. This concept is supported by recent insights in difficult-to-treat asthma, in which a greater multimorbidity burden is associated with poorer asthma control, more frequent exacerbations, impaired quality of life, and increased inflammation [42].

This supports a multidimensional and holistic approach aimed at identifying and managing clinically relevant treatable traits in patients with asthma and sinonasal diseases [24,33] (Table 1).

## 4. Pathophysiology and Phenotypes

### 4.1. One-Airway-One-Disease?

The well-established concept of the “United Airways Disease” (UAD), also referred to as “one-airway-one-disease” or “unified airways”, proposes that the upper and lower airway diseases reflect a single pathological process manifesting in different locations within the airway [43]. Historically, it is based on the consideration that the upper and lower airways are lined and linked by the respiratory epithelium, both anatomically and functionally. Similar early- and late-phase responses to airborne allergens are observed throughout the airways [44]. Over the decades, this hypothesis has been supported by basic science [45,46,47,48] and clinical [49,50,51] evidence, highlighting the shared T2 inflammatory signature. However, asthma and sinonasal diseases may result from different immune mechanisms. Three main pheno-endotypes can be described: eosinophilic atopic asthma associated with AR, the aspirin-exacerbated respiratory disease (AERD) known as Samter’s or Widal’s triad, triggered by the assumption of non-steroidal anti-inflammatory drugs, and eosinophilic asthma with CRS with or without NP [52,53,54].

A review from the Allergic Rhinitis and its Impact on Asthma (ARIA) group [55] highlighted the presence of two distinct AR phenotypes: rhinitis alone, affecting around 70–80% of patients with AR, and AR and asthma multimorbidity, affecting the remaining 20–30%. In the MeDALL (Mechanism of the Development of ALLergy) gene expression study [56], participants with rhinitis did not only express genes associated with multimorbidity but also 27 rhinitis-only genes, mostly related to Toll-like receptor (TLR)-mediated signalling, IL-17, and MyD88 (myeloid differentiation primary response gene 88) pathways. Interestingly, IL33/IL1R1 and thymic stromal lymphopoietin (TSLP), key epithelial alarmins with barrier function, appeared to be important for AR and asthma [57]; TSLP was associated with AR and asthma in children [58]. Conversely, IL-33 was not associated with rhinitis alone [59]. Moreover, DNA methylation signatures were studied in blood in childhood asthma [60]. In the MeDALL study, 14 gene sites linked to eosinophils and cytotoxic-T-cell activation were strongly associated with asthma. Differential methylation was found in 21 gene sites and shared between AR, asthma, and atopic dermatitis, with no association with a single disease [61]. In nasal brushed cells in childhood, strong DNA-methylation signatures were shared in the AR and asthma phenotype [62]. These findings, according to the authors, confirm the existence of two different diseases and suggest that the “one-airway-one-disease” model may be too simplistic and should be refined in the future.

In AERD, the diagnostic hallmark is represented by the dysregulation of lipid mediator metabolism, characterised by significantly elevated baseline levels of urinary leukotriene E4 (LTE4) and prostaglandin D2 (PGD2) metabolites, alongside blood and tissue eosinophilia; these eicosanoid levels increase during positive oral or nasal aspirin provocation testing. In contrast, AR is distinctively characterised by adaptive IgE-mediated immune-response, resulting in elevated total serum IgE and positive allergen-specific IgE titres or in vivo skin prick testing (SPT) [52].

As underlined earlier, CRS and asthma very frequently co-exist and impact the clinical outcomes of one another. Interestingly enough, the literature suggests that CRS has a greater impact on asthma than the contrary [23]. The Severe Asthma Network in Italy (SANI) reported real-life data on patients affected by severe asthma and CRSwNP, showing how comorbid patients present with higher exacerbation rate and prolonged systemic steroid use, compared to severe asthmatics without CRSwNP [13]. These data suggest that asthma and comorbid CRS represent a specific disease phenotype that requires multidimensional integration. The multidisciplinary evaluation of these patients translates into a personalised treatment approach, starting from the management of local treatments such as nasal or inhaled steroids to more advanced therapies such as biologic drugs, described below [53]. T2 inflammation underlies this association, and it is described below.

### 4.2. Type 2 (T2) Inflammation in the Airways

T2 inflammation is characterised by the predominant activity of CD4^+^ T-helper 2 (Th2), which are responsible for Th2 cytokine production [63].

In sensitized individuals, inhaled allergens are captured by antigen-presenting cells (APCs), which induce the differentiation of naïve T cells into Th2 cells and, consequently, the production and release of specific cytokines, including interleukin (IL)-4, IL-5, and IL-13. These signals drive eosinophil migration into the airways, increase mucus secretion from goblet cells, and, in particular, IL-4 and IL-13 stimulate B cells to switch from producing IgG to IgE, which then bind their receptors on basophils and mast cells, sensitizing them to the allergen [64].

The key effectors of T2 inflammation are the eosinophils, whose differentiation, activation, and recruitment from the bone marrow are primarily sustained by IL-5. These cells express receptors for IL-4 and IL-5, as well as the C-C chemokine receptor type 3 (CCR3), which binds eotaxin [65]. The eosinophil have intracytoplasmic granules containing four main cationic proteins (major basic protein, MBP; eosinophil-derived neurotoxin, EDN; eosinophil cationic protein, ECP; and eosinophil peroxidase, EPO), along with transforming growth factor β (TGF-β) and metalloproteinases, which are involved in parenchymal remodelling and fibrosis development [66].

**Figure 1:**
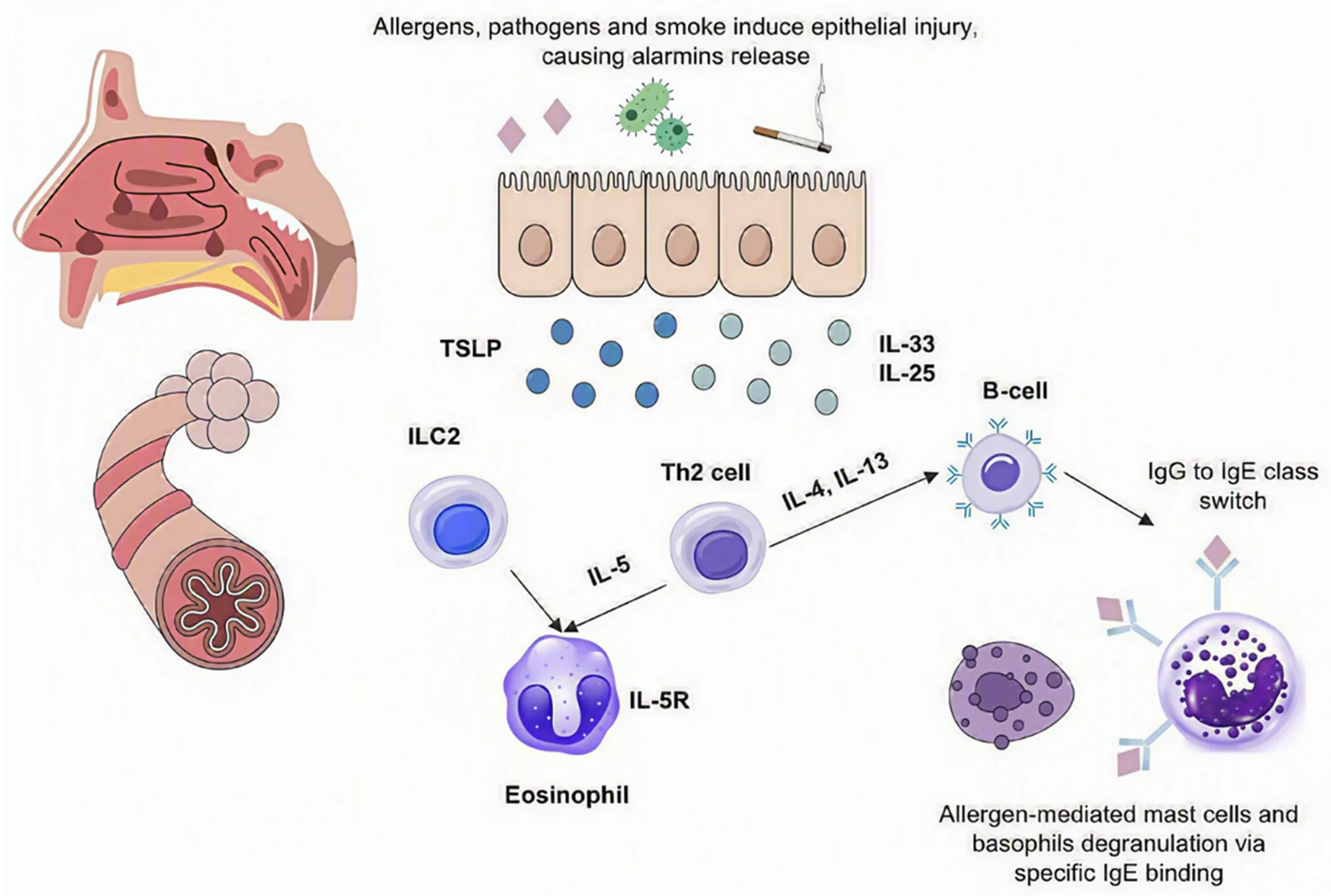
The United Airway Disease immune pathophysiology. Adapted from [68]”. Abbreviations: TSLP—thymic stromal lymphopoietin; ILC2—innate lymphoid cells; Th2—CD4+ T2-helper cell.

An additional role is played by the innate lymphoid cells (ILC2s), responsible for eosinophilic inflammation even without the clear presence of an allergen. This population is activated by the inflammatory mediators and alarmins released by the injured epithelium, such as thymic stromal lymphopoietin (TSLP), IL-25, and IL-33, which additionally enhance the ability of the APCs to present antigens to T cells. ILC2s release the characteristic T2 cytokine set, contributing to eosinophil recruitment, mucus production, and tissue repair [67]. Figure 1.

### 4.3. T2 Inflammation Biomarkers in Clinical Practice

In asthma, T2 biomarkers support the clinician during the diagnostic process and, most importantly, they are crucial for the endotyping, especially in those with severe or uncontrolled asthma who may require a biological therapy.

The atopic status of the patients can be evaluated by skin prick testing or by measuring the levels of specific IgE in serum, the latter being no more reliable and more expensive, and yet it may be preferred for spread skin disease, or if the history suggests a risk of anaphylaxis [69]. However, once an allergen is identified, the relevance of exposure and its relation to symptoms must be confirmed by the patient’s history, in order to support recommendation for allergen immunotherapy and non-pharmacological interventions (e.g., lifestyle interventions) [70]. In the context of uncontrolled severe asthma, patients who exhibit elevated total serum IgE levels may be eligible for anti-IgE therapy. Notably, baseline IgE levels do not predict the likelihood of response to anti-IgE monoclonal antibody [71].

The fractional concentration of exhaled nitric oxide (FeNO) represents the measurement in the airways of nitric oxide (NO), a short-lived gas synthetized by the NO synthase (NOS), with functions that include bronchodilation, vascular regulation, host defense, and signal transduction. Resting FeNO is low in the healthy bronchus, while the inducible isoform of NOS (iNOS), whose transcription is stimulated by IL-13 through STAT6, generates the excess signal [72]. This biomarker is higher in asthmatics with high T2 inflammation, but it may also be elevated in T2 non-asthma conditions (AR, eczema, eosinophilic bronchitis) and, conversely, not elevated in neutrophilic and obesity-related asthma [73]. FeNO is lower in smokers and during bronchoconstriction, whilst it is higher during the afternoon, and it expresses wide variability during viral respiratory infections [74].

Similar considerations can be extended to blood eosinophil count (BEC), which may be elevated in parasitic infection, atopic dermatitis, AR, CRSwNP, hypereosinophilic syndrome (HES), and eosinophilic granulomatosis with polyangiitis (EGPA). Moreover, BEC also showed an association with age, sex, time of day (higher in the morning), geographical location, obesity, and allergen exposure, and it can be decreased by the administration of nasal, inhaled, or systemic corticosteroids [75].

Under the above premises, the 2026 GINA Report [70] concludes that, in patients with typical asthma symptoms, increased FeNO levels (>50 ppb in adults and >35 ppb in children) and a BEC above the local reference range can support a diagnosis of T2 asthma, but conversely, lower levels do not rule out asthma.

Additionally, a systematic review and meta-analysis of randomised controlled trials [76] retrieved data from 6513 participants who had been randomly assigned to the control treatment arm, for which baseline FeNO and BEC were available. Higher BEC or FeNO was linked to higher asthma attack risk (per 10-fold increase, BEC relative risk—RR 1.48 [95%CI 1.30–1.68]; FeNO RR 1.44 [95%CI 1.26–1.65]), and the two combined were associated with greater risk than either factor separately [76].

In the upper airways, the role of circulating eosinophils and FeNO has been less characterized, likely due to the greater accessibility through nasal cytology. In CRSwNP patients, increased tissue eosinophil counts have been identified in cytology following nasal curettage or in fluids from nasal lavage, supporting the construct that this cell population primarily mediates the inflammatory process driving the development of polyps, either in the context of AR or non-allergic rhinitis [77]. Moreover, a strong correlation between nasal and sputum eosinophilia has been reported in adults with moderate-to-severe asthma [78]. In regard to FeNO, encouraging results were reported from a single-centre study [79] showing that FeNO levels were higher in patients with T2-CRSwNP based on histological data and that a cutoff of 27 ppb showed good performance (AUC 0.848; 95%CI 0.76–0.94) in discriminating asthmatic T2-CRSwNP.

Additional T2 biomarkers have been proposed but currently not included in clinical practice, including serum periostin, eotaxin, and thymus and activation-regulated chemokine (TARC) [80]. Briefly, periostin expression is produced via the IL-4 and IL-13 mediated activation of the STAT6 pathway, and high levels of this biomarker play a crucial role in tissue remodelling, both in upper and lower airway [81]; eotaxin and TARC bind and activate CCR3 and CCR4 chemokine receptors, respectively, which are expressed on both eosinophils and Th2 cells, thus enabling cell recruitment and activation [82]. Increased levels of these molecules have been reported in CRS compared to healthy controls, and higher levels were detected in CRSwNP than in CRSsNP [83]. Further evidence is required to understand the cost-benefit ratio of these and other upcoming biomarkers before implementing them into clinical recommendations. Table 2 summarizes the evidence provided, focusing on the clinical and therapeutical implications of the biomarkers discussed above.

## 5. Biologics for Severe Asthma and CRSwNP

Monoclonal antibodies have been demonstrated to be safe and effective in the management of severe asthma and CRSwNP. We currently have different molecules approved for both diseases, targeting different checkpoints involved in T2 inflammation and beyond. This led to a huge implementation of available data about the immunomechanisms shared between the two disorders, with T2 inflammation representing in most of the cases the cornerstone of both diseases. From a Personalised Medicine perspective, comorbidities represent a pivotal aspect when phenotyping patients. Indeed, the involvement of both upper and lower airway guides the choice for a biological therapy [84], either targeting the IL-5 or the IL-4/IL13/TSLP pathways [85]. Severe asthmatic patients are eligible for a biological treatment when the disease remains uncontrolled with maximised inhaled therapy and requires frequent or chronic systemic corticosteroid (CS) assumption, without concomitant conditions [70]. Parallelly, biological therapy represents an option for patients with severe CRSwNP when surgical approach has failed or is not indicated, and if systemic steroids are unable to control the disease [86]. Biologics are approved as add-on therapy to local inhaler or nasal corticosteroids [70].

### 5.1. Omalizumab

IgE was the first therapeutic target identified for biological treatment in atopic asthma, with omalizumab introduced in 2003 as the pioneering monoclonal antibody (mAb) in this class. Omalizumab binds to the Fc portion of circulating free IgE, thereby preventing its interaction with IgE FcεR1 receptors on effector cells. This mechanism results in reduced levels of free IgE and downregulation of receptor expression. The drug is approved for use in individuals aged 6 years and older and is administered subcutaneously every 2 or 4 weeks. The dosage is determined based on body weight and total serum IgE levels [87,88]. Omalizumab has also been demonstrated to be safe during pregnancy [89].

Patients affected by asthma and presenting with frequent exacerbations requiring systemic steroids are eligible for omalizumab if they exhibit sensitization to a perennial inhaled allergen, high total serum IgE levels, and a baseline FEV1 < 80%. In severe asthma, omalizumab has demonstrated sustained efficacy in reducing the annualised exacerbation rate (AER) by 25–61%, improving Asthma Control Test (ACT) scores, enabling significant oral corticosteroid (OCS) sparing and lowering healthcare costs [90,91,92]. In 2020, omalizumab was also shown to be effective and safe for adult patients with a diagnosis of CRSwNP. RCTs demonstrated significant, rapid, and sustained improvements in endoscopic outcomes (e.g., Nasal Polyp Score—NPS), clinical parameters, and Patient Reported Outcomes (PROs) [93].

### 5.2. Mepolizumab

Mepolizumab is an mAb that binds circulating IL-5, thereby preventing its interaction with the IL-5 receptor expressed on eosinophil and basophil surfaces. Since 2015, mepolizumab has been approved as an add-on therapy for patients with severe eosinophilic asthma characterised by frequent exacerbations requiring systemic CS and elevated BEC (≥150 cells/μL at screening or ≥300 cells/μL within the previous year). It is approved for patients aged 6 years and older and it is administered subcutaneously at a dose of 100 mg every 4 weeks [87,94].

In pivotal RCTs, mepolizumab reduced the AER by 39–53% compared with placebo in patients with severe eosinophilic asthma [95]. Treatment was also associated with improvements in FEV1, peak expiratory flow (PEF), and health-related Quality of Life (QoL) as measured by the St. George’s Respiratory Questionnaire (SGRQ) [95,96]. Post hoc analyses of the MUSCA [97] and MENSA [96] studies reported a higher treatment response rate among patients with nasal polyps (80%) compared to those without (49%). In the phase 3 SYNAPSE trial, subcutaneous mepolizumab (100 mg every 4 weeks) significantly improved the NPS and nasal obstruction Visual Analogue Scale (VAS) scores and prolonged the time to first nasal surgery compared to placebo. Based on these results, mepolizumab received approval in 2020 as an add-on therapy for CRSwNP [98]. Notably, mepolizumab is also approved for the treatment of EGPA [88] and HES at the increased dose of 300 mg administered subcutaneously every 4 weeks [99].

### 5.3. Reslizumab

Similarly to mepolizumab, reslizumab is a humanised mAb that binds IL-5, inhibiting its interaction with IL-5R. Since 2016, It has been approved as an add-on therapy for adults (≥18 years) with severe eosinophilic asthma characterised by BEC higher than 400 cells/μL. Reslizumab is administered intravenously at a dose of 3 mg/kg every 4 weeks [87,94].

Pivotal studies demonstrated that reslizumab reduced the AER by 50–59%, prolonged the time to first asthma exacerbation, and improved FEV1 values compared with baseline. Post hoc analyses also showed its capacity to reduce maintenance mOCS doses in steroid-dependent patients [100,101,102].

Evidence from RCTs and post hoc analyses showed potential benefits of reslizumab in patients with CRSwNP, particularly when coexisting with severe asthma. In a 2006 study, Gevaert et al. reported that a single intravenous dose of reslizumab was able to reduce the size of nasal polyps in 50% of patients [103]. However, data supporting its efficacy in CRSwNP remain not definitive for regulatory approval for this indication.

### 5.4. Benralizumab

Benralizumab is a humanised mAb targeting the IL-5 receptor-α, leading to direct and complete depletion of eosinophils, as well as apoptosis of basophils and ILC2 cells. A distinctive feature of benralizumab is its ability to induce anybody-dependent cellular cytotoxicity (ADCC) of the natural (NK) cells. Approved since 2017, benralizumab has been proved to be effective and safe in severe eosinophilic asthmatics presenting with frequent exacerbations requiring systemic steroid and blood eosinophilia. Benralizumab is approved for subjects older than 18 years in the EU, administered subcutaneously 30 mg every 4 weeks for the first 3 doses, followed by dosing every 8 weeks [87,94].

The two pivotal phase 3 RCTs, SIROCCO and CALIMA, demonstrated that benralizumab reduced AER by 45–51% versus placebo, depending on the dosing regimen and improved FEV1 up to 160 mL. Asthma control and asthma-related QoL consistently showed significant improvement [104,105]. In the ZONDA study, patients achieved an mOCS dose reduction of 75% [106]. Although clinical trials have shown benefits in improving NPS and other nasal outcomes, such as nasal blockage, smell impairment, time to first surgery, and SNOT-22 score compared to placebo, the U.S. FDA declined approval for its use in CRwsNP [107,108].

Notably, benralizumab is also approved for the treatment of EGPA at the dose of 30 mg every 4 weeks [109].

### 5.5. Dupilumab

Dupilumab is a fully human mAb that binds the IL-4 receptor-α subunit, inhibiting the signalling of both IL-4 and IL-13. Patients with T2 severe asthma are eligible if they experience frequent exacerbations requiring systemic CS or maintenance OCS and exhibit elevated T2 biomarkers such as BEC ≥ 150 and ≤ 1500 cells/μL or FeNO ≥ 25 ppb.

Approved since 2018, dupilumab is licensed for treatment in patients aged older than 6 years. It is administered subcutaneously at an initial dose of 400 mg followed by 200 mg every two weeks, or 600 mg initially, followed by 300 mg every two weeks in OCS-dependent severe asthmatics and in patients with coexisting moderate-to-severe T2 comorbidities, including CRSwNP or atopic dermatitis [87,94].

In the LIBERTY ASTHMA QUEST study, the risk reduction in annual AE was 47.7% and 46% with 200 mg and 300 mg, respectively. Alongside, improvements in QoL, symptom control (Asthma Control Questionnaire [ACQ]-5 and Asthma Quality of Life Questionnaire [AQLQ]), and lung function (mean FEV1 improvement up to 340 mL) were reported [110,111]. The subgroup of patients with baseline eosinophils ≥ 300 cells/μL showed greater results in both AE and lung function. In the LIBERTY ASTHMA VENTURE study, the reduction in mOCS use was approximately 70% vs. 42% in placebo [112]. Dupilumab is also approved for CRSwNP, where it significantly improved the NPS, nasal congestion/obstruction score, and sinus computed tomography (CT) score [113], as well as sense of smell [114]. Moreover, dupilumab reduced the need for sinonasal surgery, and systemic CS use [115]. Dupilumab is also approved for the treatment of atopic dermatitis, prurigo nodularis, and eosinophilic oesophagitis [116].

### 5.6. Tezepelumab

Tezepelumab is an mAb targeting the TSLP, a key epithelial alarmin involved in barrier functions. By preventing TSLP from binding to its receptor, tezepelumab acts upstream in the inflammatory cascade, leading to downregulation of key T2 cytokines including IL-4, IL-5, and IL-13. Importantly, tezepelumab has shown efficacy across the full spectrum of severe asthma phenotypes, including T2-low inflammation. Tezepelumab is currently approved for patients aged ≥12 years with severe, uncontrolled asthma and frequent exacerbations, administered subcutaneously at a dose of 210 mg every four weeks [87,94].

The PATHWAY and NAVIGATOR RCTs demonstrated up to 71% reduction in severe asthma exacerbations, alongside longer time to first exacerbation, improvements in QoL, symptom control (ACQ-6 and AQLQ), and lung function (mean FEV1 improvement up to 230 mL). To note, better benefits were observed in patients with elevated baseline T2 biomarkers (e.g., blood eosinophils and FeNO) [117,118]. In the SOURCE study, a significant improvement in oral corticosteroid dose reduction with tezepelumab was observed only in patients with BEC ≥ 150 cells [119].

The recent phase III WAYPOINT study showed that tezepelumab significantly reduced the nasal polyp size, the severity of nasal congestion, and sinonasal symptoms scores, while decreasing the need for surgery and systemic glucocorticoids compared with placebo in adults with severe, uncontrolled CRswNP [120]. In October 2025, tezepelumab was approved by the FDA and EMA for the treatment of severe CRSwNP.

### 5.7. Depemokimab

Depemokimab is a human mAb targeting circulating IL-5, similar to mepolizumab and reslizumab. It has been approved by the FDA (December 2025) and EMA (February 2026) as an add-on therapy for adults and adolescents (≥12 years) with severe eosinophilic asthma and for the treatment of severe CRSwNP. Depemokimab is the first long-acting biologic strategy administrated subcutaneously at a dose of 100 mg every 6 months [87,94].

Phase 3a SWIFT-1 and 2 studies demonstrated that depemokimab was effective in reducing the AER in severe eosinophilic asthmatic in the treatment arm (annualised rate 0.46 vs. 0.11 and 0.56 in the placebo group within the two studies) [121]. Additionally, in the ANCHOR-1 and 2 studies depemokimab showed superiority over placebo in improving the NPS and the nasal obstruction verbal response scale in patients affected by severe uncontrolled CRSwNP [122,123].

All biological agents and key results from the main RCTs for both asthma and CRSwNP are summarised in Table 3.

### 5.8. Towards a Tailored Approach for UAD

Biological therapy for severe asthma is guided by phenotypes (i.e., clinical presentation, respiratory and non-respiratory comorbidities, age of onset, etc.) and biomarkers such as BEC, IgE, and FeNO, whereas in uncontrolled CRSwNP, even if systemic and local inflammatory biomarkers are included in the patient’s evaluation, only the severity of the disease is key to initiating a biologic. For these reasons, the concomitant presence of the two diseases is crucial in the decision-making process [84].

The lack of head-to head RCTs comparing different biologics for asthma indication limits the standardization of the treatment strategies in patients eligible for more than one treatment option. Consequently, understanding the underlying driver inflammation and clinical characteristic of the patients remains crucial. The presence of non-respiratory T2 comorbidities further supports the selection process: for instance, when a patient presents with additional indications such as atopic dermatitis, eosinophilic esophagitis or chronic urticaria, the biologic approved for both diseases represents the best option. However, to date, no validated guidelines exist on strategic selection or switching of biologics for comorbid CRS and asthma. In general, for asthma and CRSwNP, mepolizumab, dupilumab, tezepelumab, and soon depemokimab may offer the greatest dual benefit for the upper and lower airways in comorbid patients. To improve our current understanding, dupilumab was recently demonstrated to be superior to omalizumab in patients with severe CRSwNP and coexisting asthma in the head-to-head EVEREST trial [125]. Furthermore, in a comparative meta-analysis evaluating the efficacy of biologics for uncontrolled asthma and CRS with or without NP, a significantly greater improvement on all outcomes in patients with CRS than those without was found [126]. In particular, tezepelumab reported the most benefit in AER reduction and symptom improvement, whilst benralizumab exhibited the greatest improvement in FEV1. Mepolizumab was associated with nasal score (i.e., SNOT-22) improvement [126]. These findings represent important insights for optimising therapeutic outcomes in clinical practice. Regarding the cumulative effect of biologics UAD, a very recent scoping review highlighted how in asthma and CRS comorbidity with or without AERD/NERD a success rate of 74% is expected. To note, dupilumab was the most widely used. Anti-eosinophilic biologics were also consistently successful in improving lower airway outcomes, but they were less performant in improving upper airway outcomes when compared to dupilumab [52].

Finally, if disease remains uncontrolled after initiating one biologic, early switching may result in better clinical outcomes. Switching biologics in patients with comorbid severe asthma and CRS should again be guided by pheno-endotypes, including the evaluation of concomitant conditions [127]. There is little evidence available on the effect of dual biologics in severe asthma or severe CRSwNP [128], although it has not been studied in clinical trials nor in patients with comorbid severe asthma and CRS.

## 6. Challenges and Future Directions

In the light of the availability of such advanced therapies, a multidisciplinary assessment involving both ENTs and pulmonologists should be considered as the Gold Standard of care. This interplay of experts should take place whenever a patient presents with the two conditions or there are suggestive signs of the presence of T2 comorbidities. This collaboration is pivotal for an optimal patient phenotyping, shifting the focus from individual disease to the underlying inflammatory pathways driving these disorders [129]. To enable the accurate identification of the correct pheno-endotype and timing for biologic initiation, more rigorous algorithms are urgently needed, particularly in the presence of multiple treatment options. Clinicians are currently highly experienced in the management of T2 diseases; however, the potential role of artificial intelligence and machine-learning should be studied in the near future.

Despite these major advancements, the multifaced role of the currently available biomarkers across different anatomical compartments may limit their interpretability. Indeed, systemic biomarkers such as BEC and IgE do not always reflect the local inflammatory activity. This is particularly true for patients under biological treatments, for whom certain biomarkers are suppressed due to drug activity [130]. Evaluating the underlying disease activity is crucial to assess the treatment efficacy during treatment. Directly assessing the local inflammation still remains challenging, in particular for asthma. This compartmental dissociation complicates accurate pheno-endotyping, precise biologic selection, and reliable treatment monitoring.

Earlier treatment initiation is currently a matter of discussion. In CRSwNP, the goals are to minimise the number of surgeries required before initiating biologic therapy and to ensure earlier access to biologics when surgery is not representing the best choice [131]. In asthma, it is important to timely identify those patients who are most likely to benefit from biological therapy, thereby preventing irreversible damage caused by chronic inflammation and long-standing disease [132]. This approach may influence not only the efficacy but also the chances of achieving disease remission [133].

Furthermore, one of the current challenges in both diseases is ensuring long-term symptom control, and, ultimately, exploring the possibility of tapering background treatments (e.g., inhaled steroids and nasal corticosteroids). In asthma, tapering ICS maintenance doses in patients treated with benralizumab has been shown to be possible while maintaining asthma control [134]. Similarly, the OPTIMAL study served as a proof of concept for algorithm titration of anti-IL-5 biologics in patients with severe asthma with clinical control on treatment [135]. Further studies on de-escalating local or biological therapies are needed to provide a framework for individualising dosing intervals. The new frontier in asthma and subsequently in CRSwNP is also currently represented by extended-interval administration of biologics, of which depemokimab will be the first one.

It is important to note that despite the high incidence of asthma and CRS worldwide, the availability of biologics is largely restricted to high-income countries, due to their elevated costs [136]. On considering that, extended-interval administration, biologic titration, and the recent introduction of biosimilars could help reduce global healthcare disparities and economic burden [137].

## 7. Conclusions

Severe asthma and CRS share multiple anatomical, physiological, and immunological mechanisms, as well as frequent respiratory and non-respiratory comorbidities. The advent of biologics has revolutionized the management of both diseases, enabling treatment through the targeting of shared immunopathological pathways. This is crucial in a Personalised Medicine approach, aimed at improving patient phenotyping based on treatable traits, thereby optimising therapeutic outcomes in clinical practice. Further head-to-head studies are needed to better understand which biologics are most likely to act synergistically on both diseases. Additionally, long term data on emerging molecules are required to strengthen and expand current knowledge on the diseases’ pathophysiology.

## Figures and Tables

**Table 1:** Comorbidities associated with asthma and chronic rhinosinusitis (CRS).

Comorbidity	Association with Asthma	Association with CRS	Clinical/Pathophysiological Link
Obstructive sleep apnoea (OSA)	Yes	Yes	Asthma and CRS may predispose to OSA through upper-airway inflammation and remodelling; conversely, OSA-related intermittent hypoxia and mechanical vibration may worsen systemic and local inflammation [22,23,24].
Vocal cord dysfunction (VCD)	Yes	Reported	Asthma and CRS-related airway inflammation and post-nasal drip may promote laryngeal dysfunction; conversely, VCD may mimic or amplify respiratory symptoms [24,26].
Dysfunctional breathing	Yes	Limited/unclear	Altered breathing patterns may amplify respiratory symptoms in asthma; its relationship with CRS remains poorly defined [26,27].
Chronic obstructive pulmonary disease (COPD)	Yes	Reported	T2 low inflammation, driven by smoking or environmental exposures, may lead to persistent airflow obstruction and the coexistence of COPD and asthma; oxidative stress and systemic inflammation may link COPD with CRS [28,29].
Bronchiectasis	Yes	Reported	Persistent airway inflammation may promote epithelial damage and impaired mucociliary clearance, favouring bronchiectasis; sinonasal inflammation may act as a reservoir for respiratory pathogens [2,31,32].
Gastroesophageal reflux disease (GERD)	Yes	Yes	Acid reflux and microaspiration may promote bronchoconstriction and sinonasal mucosal irritation, worsening respiratory symptoms and disease control in both asthma and CRS [21,24,27,34].
Obesity	Yes	Yes	Obesity may worsen asthma and CRS through systemic adipose inflammation; it may also reduce corticosteroid responsiveness [21,27].
Type 2 diabetes mellitus (DMII)	Yes	Yes	Hyperglycaemia-related impairment of innate immunity may favour microbial colonisation and worsen CRS, while the pathophysiological link between DMII and asthma remains unclear [27].
Anxiety and depression	Yes	Yes	Asthma and CRS-related symptom burden may favour anxiety and depression through serotonin dysregulation, systemic inflammation, genetic or environmental susceptibility, and corticosteroid neurotoxicity [21,27,40].

**Table 2:** Summary of evidence on established and emerging T2 biomarkers.

Biomarker	Biological Significance	Clinical Context or Population	Clinical/Therapeutical Implications
Specific serum IgE	Atopic status Relevance of exposure and its relation to symptoms must be clinically confirmed [70].	General population	Lifestyle interventions (e.g., avoiding the trigger) Consider allergen-specific immunotherapy based on clinical relevance. Eligibility to anti-IgE Ab [70].
Total serum IgE	Atopic status	T2 Asthma CRS Anaphylaxis	Elevated level required to consider anti-IgE therapy [71].
FeNO	IL-13 (via STAT6)-mediated stimulation of iNOS, reflecting T2 inflammation [72]. Reduced in smokers and bronchoconstriction; wide variability during viral respiratory infections [74].	T2 asthma CRS	FeNO levels >50 ppb in adults and >35 ppb in children can support a diagnosis of T2 asthma, but conversely, lower levels do not rule it out [70]. FeNO levels >27 ppb discriminated asthmatic T2-CRSwNP [79]. Eligibility to dupilumab [70].
BEC	Eosinophilic inflammation Higher in the morning, influenced by geographical location, obesity, and allergen exposure. Decreased under corticosteroids [70].	T2 asthma and non-asthma conditions; HES; EGPA; parasitic infections	BEC above the local reference range can support a diagnosis of T2 asthma, but conversely, lower levels do not rule out asthma [70]. Eligibility to anti IL-5/IL-5R Ab [70]
Periostin	IL-4 and IL-13-mediated activation of the STAT6 pathway High levels responsible for tissue remodelling in upper and lower airway [81].	Increased levels in CRS vs. healthy controls, and higher levels in CRSwNP than in CRSsNP [83].	Further evidence needed to understand the cost-benefit ratio of these emerging biomarkers, before implementing them into clinical recommendations [83].
Eotaxin	Binds and activates CCR3 on eosinophils and Th2 cells, driving cell recruitment and activation [82].		
TARC	Binds and activates CCR4 on eosinophils and Th2 cells, driving cell recruitment and activation [82].		

List of abbreviations: ABPA: allergic bronchopulmonary aspergillosis; Ab: antibodies; IgE: immunoglobulin E; CRSwNP: chronic rhinosinusitis with nasal polyps; FeNO: fractional concentration of exhaled nitric oxide; iNOS: inducible nitric oxide synthase; BEC: blood eosinophil count; HES: hypereosinophilic syndrome; EGPA: eosinophilic granulomatosis with polyangiitis; CCR: cytokine and chemokine receptor; TARC (thymus and activation-regulated chemokine).

**Table 3:** Results from randomised controlled trials of biological drugs in the treatment of severe uncontrolled asthma and CRSwNP.

	Pivotal Studies on Asthma	Main Outcomes	Pivotal Studies on CRSwNP	Main Outcomes
Anti-IgE Omalizumab	Busse, 2001 Solèr, 2001 INNOVATE, 2005 [90,91,92]	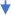 AER 25–61% 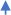 QoL 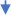 ICS dose ≥ 50%	POLYP 1 and POLYP2, 2020 [93]	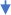 NPS and NCS 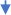 SNOT-22 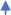 QoL
Anti-IL-5 Mepolizumab	DREAM, 2012 (i.v) MUSCA, 2017 (s.c) MENSA, 2014 (s.c) [95,96,97]	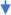 AER 39–53% 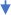 Time to 1st AE 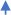 FEV1L and PEF 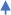 QoL	SYNAPSE, 2021 [98]	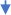 NPS and nasal VAS 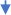 SNOT-22 and surgery/systemic steroids requirement.
Anti-IL-5 Reslizumab *	Castro, 2015 Bjermer, 2016 Corren, 2016 [100,101,102]	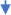 AER 50–59% 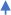 FEV1L 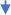 Time to 1st AE 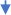 mOCS	Gevaert, 2006 [103]	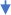 Size of polyps
Anti-IL-5R Benralizumab *	SIROCCO AND CALIMA, 2016 ZONDA, 2017 [104,105,106]	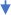 AER up to 51% 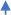 FEV1L 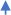 QoL 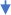 mOCS	OSTRO, 2024 and ORCHID, 2022 [107,108]	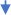 NPS and nasal blockage score, CT score, SNOT-22, NRS
Anti-IL4/13R Dupilumab	Wenzel, 2016 LIBERTY ASTHMA QUEST and VENTURE, 2018 [110,111,112]	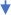 AER up to 87% 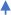 FEV1L 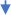 ACQ-5 and AQLQ 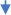 mOCS	LIBERTY SINUS-24 and 52, 2019 [113]	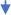 NPS and CT score 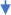 SNOT-22
Anti-TSLP Tezepelumab	PATHWAY, 2017 NAVIGATOR, 2020 SOURCE, 2022 [117,118,119,124]	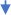 AER 62–71% 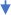 Time to 1st AE 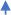 FEV1 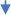 ACQ-6 and AQLQ 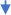 mOCS (if BEC ≥ 150 cells/μL)	WAYPOINT, 2025 [120]	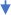 NPS and NCS 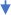 SNOT-22, loss of smell score, need for surgery, total symptoms
Long-acting anti-IL-5 Depemokimab	SWIFT 1 and 2, 2025 [121]	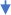 AER	ANCHOR 1 and 2 [122]	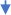 NPS and NCS 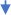 nasal obstruction verbal response scale score

Abbreviations: Asthma Exacerbation (AE); Annualised Exacerbation Rate (AER); Quality of Life (QoL); Nasal Polyp Score (NPS); Nasal Congestion Score (NCS); Visual Analogue Scale (VAS); Sino Nasal Outcome Test-22 (SNOT-22); Forced Expiratory Volume in the 1st second (FEV1), Computed Tomography (CT), Asthma Control Questionnaire (ACQ), Asthma Quality of Life Questionnaire (AQLQ); Blood Eosinophil Count (BEC). * not approved for CRSwNP.

## Data Availability

No new data were created or analyzed in this study. Data sharing is not applicable to this article.
